# Effect of Ketogenic Diet on Quality of Life in Adults with Chronic Disease: A Systematic Review of Randomized Controlled Trials

**DOI:** 10.3390/nu13124463

**Published:** 2021-12-14

**Authors:** Myriam Abboud, Fatme AlAnouti, Evridiki Georgaki, Dimitrios Papandreou

**Affiliations:** 1 Department of Health, College of Natural and Health Sciences, Zayed University, Dubai 19282, United Arab Emirates; myriam.abboud@zu.ac.ae; 2 Department of Health, College of Natural and Health Sciences, Zayed University, Abu Dhabi 144534, United Arab Emirates; fatme.alanouti@zu.ac.ae; 3 Barts and the London School of Medicine and Dentistry, Queen Mary University of London, London E1 4NS, UK; evridiki.georgaki@outlook.com

**Keywords:** diet, ketogenic, quality of life, chronic disease, systematic review, meta-analysis

## Abstract

Background: Chronic diseases adversely affect quality of life (QOL). The ketogenic diet (KD) may improve the QOL. Objective: The aim of this systematic review was to summarize the available evidence of randomized controlled trials (RCTs) to establish the effect of KD on the QOL in adults with chronic diseases. Methods: Reporting followed PRISMA guidelines. We included randomized controlled trials (RCTs) conducted on adults with chronic disease including an intervention group that received KD and a control group, and where QOL was reported as outcome. We searched PubMed, APA PsycInfo, EMBASE, the Cumulative Index to Nursing and Allied Health Literature (CINAHL), the Cochrane Library, and Clinicaltrials.gov, and the references of the included articles and previous relevant reviews, without language or time restrictions. We critically appraised included studies and narratively synthesized their findings. Results: Nine RCTs were included. The risk of bias was low, except of allocation concealment and blinding. In patients with cancer: one RCT found an improvement in overall QOL, another reported improved physical component summary, and one found no superiority of KD in all QOL domains. In patients with neurological disorders: improved QOL was reported in Alzheimer’s disease patients, whereas no difference in mental and physical health QOL was noted in patients with multiple sclerosis. In patients with obesity and type II diabetes: one RCT reported superiority of energy-restricted KD in improving role functioning, mental health, health perceptions, and pain compared with guideline-based diet, whereas in another RCT, high and low carbohydrate diets achieved comparable improvements. Among patients with knee osteoarthritis, no differences between KD and low-fat groups were noted. Dietary compliance with the KD, reported in three studies, was shown to be high. Side effects were mostly noted during the first weeks of intervention, and adverse events were not markedly different with KD and the comparison diet. Conclusions: The evidence from RCTs investigating the effect of KD on QOL in adults with chronic disease is inconclusive. The promising effect noted in some included studies and the low rates of adverse events and side effects encourage future investigations in this regard.

## 1. Introduction

Chronic diseases are health conditions characterized by long-term physical and/or mental impairments requiring lengthy periods of supervision and care [1,2]. Professional bodies vary in the use of the term chronic, in addition to the variation in the diseases included under its umbrella [2,3]. According to the World Health Organization (WHO), a chronic disease is defined as being of long duration, slow progression, and non-transmissible [4].

Quality of life (QOL) is a concept approachable at varying levels of generality and the definitions are diverse [5]. It is the individuals’ insights of their position in life relative to their goals and expectations [6], and its concept encompasses all domains of life including the psychological, social, and economic well-being of individuals, and their relationships to relevant features of the environment [7]. QOL is best understood as representing the gap between one’s actual functional level and one’s ideal standard [8]. As strongly associated with morbidity, disability, and mortality [9,10,11], chronic diseases affect the QOL by threatening the physical and emotional well-being, and through the development of chronic stress [12,13,14]. QOL is hence an important outcome in chronic health conditions [15], complimenting the traditional evaluation of reducing morbidity and mortality [16].

Although there is no standardized definition of the ketogenic diet (KD) [17], it is characterized in general by a reduction in carbohydrates (CHO) and relative increases in the proportions of proteins and fats, enabling an increased utilization of ketones in the body [18,19]. The main types of the KD include the traditional ketogenic diet (TKD) containing a fixed ratio by weight of fat to combined protein and CHO [20], the medium-chain triglyceride (MCT) KD using MCT oil to provide around half the calories [21], and the modified Atkins diet (MAD) [22]. The clinical importance of these diets began with their successful use in the treatment of intractable childhood epilepsy [23]. Furthermore, ample evidence supports the broader therapeutic actions and effectiveness of the use of these diets in the improvement of some metabolic pathways such as cancer, type two diabetes (T2D), cardiovascular diseases (CVDs), polycystic ovary syndrome (PCOS), and some neurological disorders, leading to beneficial health effects [23,24,25]. However, there are still some concerns regarding their potential adverse effects including micronutrient deficiencies, appetite reduction, nausea, constipation, fatigue, hyperlipidemia, and unintended weight loss [19,23]. KD may improve the QOL by reducing chronic pain, inflammation, and improving metabolic parameters through multiple mechanisms [26]. The ketone bodies produced by the liver results in a greater production of ATPs with a potential increase in available energy [27], reduction in the production of reactive oxygen species [28], and inhibition of pro-inflammatory cytokine mediators [29], although conclusive evidence in this regard is lacking.

The aim of this systematic review is to summarize the available evidence of randomized controlled trials (RCTs) to establish the effect of KD on the QOL in adults with chronic diseases.

## 2. Materials and Methods

### 2.1. Review Design

This systematic review was conducted following a predefined protocol that was registered at the OSF registries (DOI: 10.17605/OSF.IO/2MK5G). The reporting of the literature searching component of the systematic review was conducted according to the Preferred Reporting Items for Systematic reviews and Meta-Analyses literature search extension (PRISMA-S) [30], and that of the systematic review according to the PRISMA statement [31]. Ethical approval for this study was not required.

### 2.2. Criteria for Study Inclusion

The inclusion criteria were designed according to the Population, Intervention, Comparator, Outcome, and Study design (PICOS) principle. Accordingly, randomized controlled trials (RCTs) thath have been conducted on adults with chronic disease; including an intervention group and received KD and a control group and assessed QOL as an outcome were included. Regarding the population, although there is a large variation in the use of the term “chronic disease” [32], studies reporting on chronic disease, defined as a disease that is long in duration, has a slow progression, and is not passed from person to person [33] were included. Studies reporting on adult patients, as defined by the investigators (e.g., aged > 18 years at baseline) were included. Regarding the intervention, although there is no standardized definition of the KD, studies reporting on diets high in fat, low in CHO resulting in hyperketonemia [17] such as classical KD, medium-chain triglyceride (MCT)-KD, and MAD were included. When the intervention was not specified as a KD, an upper limit of 50 g of CHO per day or 10% energy from CHO [34] was retained for inclusion. RCTs involving a co-intervention were included if both arms of the study received the same co-intervention. Regarding the comparator, studies employing any other type of diet (e.g., low fat diet, anti-inflammatory diet, high fiber diet, or a different form of KD) were included. Regarding the outcome, studies reporting on QOL were included irrespective of the definition adopted, nor of the assessment tool. Finally, regarding study design, only RCTs were included, irrespective of blinding.

Studies were excluded if they were conducted on healthy adults, adults with acute conditions, or on pediatric participants; were conducted on non-human species; were non-randomized or non-controlled; or were reported on in non-original articles without detailed empirical data such as posters, conference abstracts, book chapters, or reviews.

### 2.3. Search Strategy

The search strategy was validated by a medical librarian. The search involved two key concepts: (1) KD and (2) QOL. For each concept, controlled vocabulary such as Medical Subject Headings (MeSH) terms and keywords were mapped. Search terms included but were not limited to quality of life or well-being, combined with keto or Atkins. PubMed, APA PsycInfo via Ovid, EMBASE via Ovid, the Cumulative Index to Nursing and Allied Health Literature (CINAHL) via EBSCO, the Cochrane Library, and Clinicaltrials.gov were searched, without any language or publication date restriction. The literature search was conducted on 16 October 2021 by one author (MA), and the electronic search strategy run on Embase via Ovid and PubMed is available in Supplementary Material Table S1. Bibliographies of the included articles and relevant reviews were also hand-searched for eligible studies.

### 2.4. Study Selection

Two sets of authors (MA/FA; EG/DP) screened titles and/or abstracts retrieved by the search independently and in duplicate, and identified studies that potentially met the inclusion criteria. Then, the full texts of potentially eligible studies were retrieved and assessed independently and in duplicate for eligibility. Study selection was conducted following calibration exercises to ensure the validity of the process. Disagreements were solved through discussions or, in the absence of a consensus, with a third reviewer.

### 2.5. Data Extraction

The same two pairs of authors (MA/FA; EG/DP) extracted data from eligible studies, independently and in duplicate, using a data extraction form, and following a calibration exercise to ensure the validity of the process. For all included articles, the authors extracted the characteristics of the study, details of the population included (number of enrolled patients, demographic characteristics, type of chronic disease), intervention (features of KD, duration of intervention, compliance), comparator, and outcomes (QOL, adverse events, and attrition. When studies were lacking information, original references were retrieved for additional data on the design and results.

### 2.6. Quality Assessment

The same two pairs of authors (MA/FA; EG/DP) assessed the risk of bias of included studies, independently and in duplicate, following the Cochrane criteria (sequence generation, allocation concealment, blinding of participants and outcome assessors, incomplete outcome data, and selective outcome reporting) [35]. For each study, each potential source of bias was graded as low, high, or unclear risk. Disagreements were solved through consensus or with the help of a third reviewer.

### 2.7. Data Synthesis

As a meta-analysis was not possible, the author provided a narrative synthesis of the findings from the studies including the author-recorded characteristics of the study, details of the population included, the intervention, the comparator, the assessment methods and tools as well as the study’s outcomes.

## 3. Results

### 3.1. Search Results

**Figure 1:**
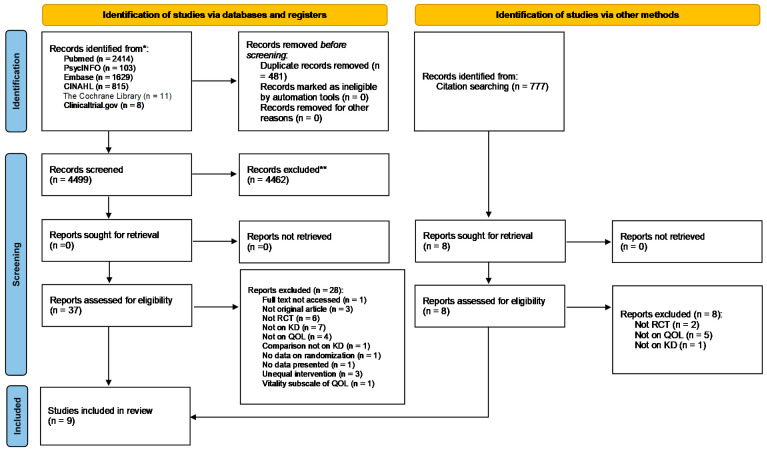
Preferred Reporting Items for Systematic Reviews and Meta-Analyses (PRISMA) diagram of study selection. CINAHL: Cumulative Index to Nursing and Allied Health Literature; RCT: Randomized Controlled Trial; KD: Ketogenic Diet; QOL: Quality of Life.

Results of the study selection process are displayed in Figure 1. The initial search resulted in 4980 screened records, out of which nine RCTs were included in this review.

### 3.2. Characteristics of Included Studies

Characteristics of the included RCTs are summarized in Table 1 and detailed in Supplementary Material Table S2. Three studies were conducted in the USA [36,37,38], and one study took place in each of Trinidad and Tobago [36], Australia [37], Canada [38], UK [39], Iran [40], and in New Zealand [41]. Most of the studies were published within the last two years [37,38,39,41,42,43,44].

Regarding chronic diseases, four studies were conducted on cancer patients (several types of stages 2 and 3 cancer [36], ovarian or endometrial cancer [42], glioblastoma [39], and breast cancer [40]), two on patients with neurological disorders (multiple sclerosis [43] and Alzheimer’s disease [41]), two on patients with obesity, and type II diabetes [37,38], and one on patients with knee osteoarthritis [44]. Regarding the design, only the study by Philips et al. [41], was a two-period crossover RCT.

The prescription of the KD varied between studies including a CHO intake less than 20 g per day [42,44], or less than 50 g per day [37,38] or net CHO less than 6% of total energy per day [41]. Only in Durrer et al. [38] did the intervention consist of a commercial ketogenic weight loss diet plan supplemented with whole foods; whereas in Augustus et al. [36], Lee et al. [43], and Martin McGill et al. [39], the intervention consisted of MCT-KD. The duration of intervention ranged between 12 weeks [38,40,41,42,44] and 12 months [37]. Achievement of ketosis was measured either by blood ketones [36,37,39,40,41,42,43,44], mainly beta-hydroxybutyrate, or urinary ketones [36,39]; whereas only Strath et al. [44] did not assess ketosis. The comparators varied between standard traditional diet [36,40], energy-restricted, high CHO diet [37], the American Cancer Society diet [42], Diabetes Canada diet [38], modified Paleolithic diet [43], low fat diet [44], and usual diet [41,43,44]. Only Martin McGill et al. [39] compared two forms of the KD: MCT-KD and the modified MKD.

QOL was assessed using a variety of tools including disease-specific questionnaires such as the European Organization for Research and Treatment of Cancer current core questionnaire (EORTC QLQ-C30) [36,39,40], the Diabetes-39 questionnaire [37], the Multiple Sclerosis Quality of Life-54 questionnaire [43], the Quality of Life in AD questionnaire [41], and the Knee Injury and Osteoarthritis Outcome Score quality of life questionnaire [44] or generic questionnaires such as the Medical Outcomes Study Short Form (SF) [38,42].

**Table 1:** Characteristics of the included studies.

First Author, Year, Country	Study Population & Type of Chronic Disease	Age; %Male	Duration	Intervention: Features of KD	Control	Isocaloric Diets (arms)	Co-intervention	Assessment of Ketosis	Assessment of QOL
Cancer									
Augustus, 2021, Trinidad and Tobago (Trinidad) [36]	Stages 2 and 3 cancer patients, receiving chemotherapy or radiation, nonvegetarian, on a CHO-based diet (>40%) I: *n* = 20; 16 completers C: *n* = 20; 20 completers	Age: mean (SD): I: 49.80 ± 6.72 C: 51.80 ± 4.18 %Male: NR	16 weeks	MKD: 7-day cyclic altered KD (≈10% CHO (50 g), 15% Protein (75 g), 75% Fat (167 g); 2000 Kcal); main source of Fat: MCT	Standard traditional diet	Not specified by study protocol	None	Urinary ketones: dip stick test and urine analyzer	EORTC QLQ-C30
Cohen, 2018, Birmingham (USA) [42]	Women with ovarian or endometrial cancer, BMI ≥ 18.5 kg/m^2^ I: *n* = 37; 25 completers C: *n* = 36; 20 completers	Age: mean (SD): I: 61.5 ± 8.5 C: 58.6 ± 11.7 %Male: 0%	12 weeks	KD: 5% CHO (≤20 g); 25% Protein (≤100 g); 70% Fat (≤125 g)	ACS diet	Neither group was instructed to alter total energy intake	None	Serum BHB: SIRRUS analyzer Urinary ketones: strips	SF-12 (PCS and MCS)
Khodabakhshi, 2020, Tehran (Iran) [40]	80 women with locally advanced or metastatic breast cancer receiving chemotherapy for ≥12 weeks I: *n* = 40; 30 completers C: *n* = 40; 30 completers	Age Range: 18–70 I: 44.8 ± 8.4 C: 45.2 ± 15.0 %Male: 0%	12 weeks	6% CHO, 19% Protein, 20% MCT, 55% Fat	55% CHO, 15% Protein, 30% Fat	Both diets calculated to be eucaloric	None	Blood BHB: home kit	EORTC QLQ-C30 and EORTC QLQ-BR23
Martin-McGill, 2020, United Kingdom [39]	12 patients with glioblastoma planning to go temozolomide chemotherapy and radiotherapy MKD: *n* = 6; 1 completed 12 weeks; 1 completed 12 months MCTKD: *n* = 6; 3 completed 12 weeks; 2 completed 12 months	Age Median: 57; Range: 44–66 %Male: 66.60%	12 weeks 12 months	I1: MKD: 5% CHO, 80% Fat, 15% Protein I2: MCTKD: 10% CHO, 75% Fat (30% from MCT nutrition product), 15% Protein	None	Not specified by study protocol	None	Urinary ketones: dip stick test Blood ketones: home kit	EORTC QLQ C30 and BN20
Neurological disorders									
Lee, 2020, Iowa (USA) [43]	15 patients with relapsing remitting multiple sclerosis or progressive relapsing-remitting multiple sclerosis (expanded disability status ≥ 4.5) KD: *n* = 5; 4 analyzed (1: insufficient data) MPD: *n* = 6 Usual diet: *n* = 4	Age Total: Range: 36–63 Mean (SD): 51.9 ± 9.5 KD: 51.8 ± 11.8 MPD: 50.3 ± 9.5 C: 54.5 ± 11.8 %Male: 50%	12 weeks	MCT-based KD: ketogenic version of the modified Paleolithic diet with supplemental MCTs to achieve a daily goal of 70% of total Kcal from fat)	Modified Paleolithic diet C: Usual diet	Not specified by study protocol	Pre-study vitamins, supplements, and/or medications	Plasma BHB: NR	Multiple Sclerosis Quality of Life-54
Philips, 2021, Hamilton (New Zealand) [41]	26 patients with Alzheimer diseases BMI > 18.5 kg/m^2^ Phase 1 KD: *n* = 13; 11 completers Usual diet: *n* = 13; 13 completers Phase 2 KD: *n* = 13; 10 completers Usual diet: *n* = 13; 13 completers	Age Total: Range: 57–79 Mean (SD): 69.8 ± 6.0 KD > Usual diet: Range: 57–77 Mean(SD): 68.0 ± 5.4 Usual diet > KD: Range: 61–79 Mean(SD): 71.7 ± 6.2 %Male: Total: 62% KD > Usual diet: 77% Usual diet > KD: 46%	12 weeks: I or C 10 weeks: washout	58% Fat (26% SFA, 32% non-saturated), 29% Protein, 7% Fiber, 6% net CHO by weight	Usual diet 11% Fat (3% SFA, 8% non-saturated), 19% Protein, 8% Fiber, 62% net CHO by weight	Not specified by study protocol	Daily multivitamin	Serum BHB: ketone blood monitor	QOL-AD
Obesity and T2DM									
Brinkworth, 2016, Adelaide (Australia) [37]	Adults with T2DM (HbA1c ≥ 7.0% or taking a diabetes medication), overweight and obese (BMI: 26–45 kg/m^2^) I: *n* = 58; 41 completers C: *n* = 57; 37 completers	Age Range: 35–68 Mean (SD) I: 58 ± 72 C: 58 ± 7 %Male: I: 64% C: 51%	12 months	Very-low CHO, high-fat diet: 14% CHO (<50 g); 28% Protein, 58% Fat (35% MUFA, 13% PUFA, <10% SFA)	High-CHO, low-fat diet: 53% CHO; 17% Protein; <30% Fat (15% MUFA, 9% PUFA, <10% SFA)	For I and C: ∼30% energy restriction (500–1000 Kcal/day)	60-min, moderate-intensity, exercise: 3 days/week	Plasma BHB: D-3 Hydroxybutyrate kit	Diabetes-39
Durrer, 2021, Southern British Columbia (Canada) [38]	Adults with T2DM, using glucose-lowering medication, obese (BMI ≥ 30 kg/m^2^) I: *n* = 98; 78 completers (98 ITT) C: *n* = 90; 60 completers (90 ITT)	Age: mean (SD): I: 58 ± 11 C: 59 ± 8 %Male: I: 44% C: 43%	12 weeks	Low-CHO energy-restricted commercial weight loss plan supplemented with whole foods (<50 g CHO; 35–45 g Fat, 110–120 g Protein; 850–1100 Kcal)	Information conforming with 2013 Diabetes Canada Clinical Practice Guidelines	Not specified by study protocol	None	Capillary blood ketones: NR	SF-20
Knee Osteoarthritis									
Strath, 2020, Birmingham (USA) [44]	21 adults with knee osteoarthritis LCD: *n* = 8 LFD: *n* = 6 C: *n* = 7	Age Range: 65–75 Mean (SD) LCD: 71.00 ± 3.12 LFD: 72.33 ± 1.97 C: 68.71 ± 7.11 %Male: LCD: 60%; LFD: 75%; C: 80% (Completers: LCD: 60%; LFD: 100%; C: 75%)	12 weeks	Kcal: unlimited; Fat: unlimited; CHO: 20 g; Proteins: 100 g	LFD: Kcal: 800–1200; Fat: 50–67 g; CHO: unlimited; Proteins: 100 g C: Kcal, Fat, CHO, Proteins: unlimited	No	None	Not measured	Knee Injury and Osteoarthritis Outcome Score quality of life

ACS: American Cancer Society; BHB: B-Hydroxybutyrate; BMI: Body mass index; C: Control; CHO: Carbohydrate; EORTC QLQ-C30: European Organization for Research and Treatment of Cancer current core; GHS: Global Health Status; HbA1c: Glycated hemoglobin; I: Intervention; ITT: Intention to treat; KD: Ketogenic diet; LCD: Low-carbohydrate diet; LFD: Low fat diet; MCS: Mental component summary; MCT: Medium chain triglycerides; MCTKD: Medium chain triglyceride ketogenic diet; MKD: Modified ketogenic diet; MPD: Modified Paleolithic diet; MUFA: Monounsaturated fatty acids; NR: Not reported; PCS: Physical component summary; PUFA: Polyunsaturated fatty acids; QOL: Quality of life; QOL-AD: Quality of Life in Alzheimer’s Disease; SD: Standard deviation; SF-12: Medical Outcomes Study Short Form-12 Health Survey; SF-20: Medical Outcomes Study Short Form-20 Health Survey; SFA: Saturated fatty acids; T2DM: Type 2 diabetes mellitus; WHO: World Health Organization.

### 3.3. Assessment of Risk of Bias

**Figure 2:**
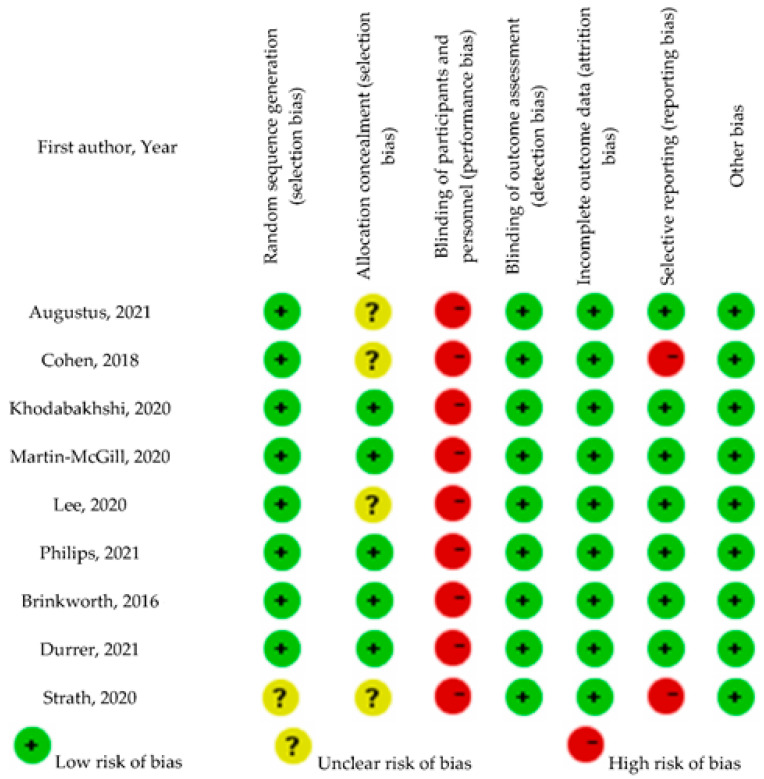
Risk of bias of included studies from consensus between a pair of raters [36,37,38,39,40,41,42,43,44].

Results of the assessment of risk of bias of the included RCTs are detailed in Figure 2. Risk of bias regarding sequence generation was low for all studies, expect for Strath et al. [44]. Allocation was concealed in Brinkworth et al. [37], Durrer et al. [38], Khodabakhshi et al. [40], Martin-McGill et al. [39], and Philips et al. [41], and was unclear in the remaining trials [36,37,38,39]. Personnel were blinded only in the studies by Brinkworth et al. [37], Lee et al. [43], and Philips et al. [41]. In general, risk of bias for blinding of outcome assessment and completeness of outcome data was low. Finally, selective reporting was suspected for the studies by Augustus et al. [36], Cohen et al. [42], Martin McGill et al. [39], and Strath et al. [44].

### 3.4. Results of Included Studies

The findings from the included studies are summarized in Table 2 and detailed in Supplementary Material Table S2. As shown in Table 2a, in patients with a diagnosis of cancer, the evidence regarding the effect of KD on QOL was inconclusive [36,39,40,42]. Augustus et al. [36] found a significant improvement in overall QOL, whereas Cohen et al. [42] reported improved QOL only at the level of physical component summary. In the study conducted by Martin McGill et al. [39], quantitative results were limited to only three patients, favoring the MKD. Finally, Khodabakhshi et al. [40] found no significant differences in the effects of KD and regular diet on all domains of QOL.

Additionally, among patients with neurological disorders, conflicting results were noted, whereby Philips et al. [41] reported improved QOL in Alzheimer’s disease patients on the KD compared with those on a usual diet, whereas Lee et al. [43] found no significant difference in mental health and physical health QOL scores among the compared groups in patients with multiple sclerosis.

Similarly, among patients with obesity and Type II diabetes [37,38], contradictory results were noted. Durrer et al. [38] showed that a ketogenic, energy-restricted diet resulted in improved measures of role functioning, mental health, health perceptions, and pain compared with a diet and lifestyle conforming with the Diabetes Clinical Practice Guidelines, whereas Brinkworth et al. [37] found that both high and low CHO diets achieved comparable improvements in QOL.

Finally, among patients with knee osteoarthritis [44], there were non-significant differences between the KD and low-fat groups after post hoc analysis.

**Table 2:** (**a**) Results of included studies. (**b**). Results of included studies.

(**a**)				
**First Author, Year**	**Effect on QOL**		**Conclusion**	
**Cancer**				
Augustus, 2021 [36]	Mean change: I: +28 (Sig.); C: +0.6 (NS) Sig. between-group difference over time; effect size: 0.268 (medium) Inverse association between urinary ketones and QOL (b = −3.175, 95% CI = −5.723, −0.626)		Keto-adapted patients on a MKD had an improvement in self-reported QOL over time KD may improve QOL of cancer patients (not inclusive of advanced stage cancer) compared with patients on a standard traditional diet	
Cohen, 2018 [42]	Sig. within-group increase in PCS in I (+11%); NS change in C Sig. between-group difference in adjusted PCS, NS between-group difference in MCS NS association between PCS or MCS and serum BHB		In women with ovarian or endometrial cancer, a KD does not negatively affect quality of life and may improve physical function	
Khodabakhshi, 2020 [40]	Mean difference (95% CI): Physical functioning: 9.9 (−0.7, 20) (NS) Role functioning: 8.9 (−6, 23) (NS) Cognitive functioning: 5.5 (−8, 14) Emotional functioning: 2 (−10, 14) Social functioning 3.5 (−4.6, 5.9) (NS) Global quality of life: 8.1 (−5.7, 3.3) (NS)		After adjusting baseline values and chemotherapy status, NS differences in all domains of QOL between I and C KD diet combined to chemotherapy in patients with breast cancer does not bring additional benefit	
Martin-McGill, 2020 [39]	Week 6 onward, GHS improved for the patient following MKD and reduced for patients following MCTKD		For retained patients at 12 months, GHS reduced within the MCTKD group and improved in the MKD group	
Neurological disorders				
Lee, 2020 [43]	NS between-group differences in mental health and physical health		NS differences in mental health and physical health QOL scores among groups Suggested clinically sig. improvements in mental health and physical health QOL with Modified Paleolithic diet (change > 5) Suggested clinically sig. decline in mental health and physical health QOL with usual diet	
Philips, 2021 [41]	Treatment effect (mean ± SD) Phase 1: KD > Usual diet: +2.86 ± 4.64; Usual diet > KD: −1.15 ± 5.41 Phase 2: KD > Usual diet: +0.31 ± 3.68; Usual diet > KD: +3.03 ± 7.52 All patients: KD > Usual diet: +2.95 ± 6.12; Usual diet > KD: −0.42 ± 4.60 Overall treatment effect: +3.37 ± 6.86 (Sig. change)		Patients on KD had improved QOL compared to those on usual diet High rates of retention and adherence are achievable in applying a 12-week MKD to patients with Alzheimer’s disease and adverse effects are mild	
Obesity and T2DM				
Brinkworth, 2016 [37]	NS between-group differences in anxiety and worry, social burden, sexual functioning, and energy and mobility		In overweight and obese adults with T2DM, both high and low CHO diets achieved comparable improvements in QOL	
Durrer, 2021 [38]	Treatment effect (95% CI): Physical Functioning: 0.7 (−7.7, 9.9) * Role Functioning: 13.6 (2.4, 26.3) * Social Functioning: 6.1 (−2, 14.3) * Mental Health: 6.9 (1.9, 12.7) * Health Perceptions: 19.2 (13.2, 25.4) (NS) Pain: −7.5 (−17.2, −0.1) * (* a precise *p*-value could not be obtained)		In obese patients with T2DM, there was sig. improvement in role functioning, mental health, health perceptions, and pain with low-CHO energy-restricted diet compared with the usual diet	
Knee Osteoarthritis				
Strath, 2020 [44]	LCD: sig. withing-group change (≈−0.6) LFD: sig. withing-group change (≈−0.2) C: sig. withing-group change (≈−0.4) NS time * diet interaction and NS differences in LCD and LFD group after post hoc analysis		NS differences in LCD and LFD group were noted after post hoc analysis	
(**b**)				
**First Author, Year**	**Compliance**	**Ketosis**	**Adverse events/Side effects**	**Attrition**
Cancer				
Augustus, 2021 [36]	Three-day food diaries (2 weekdays and 1 weekend) obtained at the weeks 6 and 12	Sig. rise in urinary ketones in I vs. C	I: side-effects related to keto-adaptation (first 2–6 weeks; sig. reduced 6 weeks post treatment): fatigue, dizziness, reduced energy C: headaches/migraines Unable to determine whether reduced energy or fatigue are attributed to I or by natural progression of the disease	I: 2% [*n* = 4: nausea and vomiting related to I affecting subjects’ palatability (*n* = 2); inability to complete testing at all follow-up times (*n* = 1); mortality not related to medical treatment nor I (*n* = 1)] C: 0%
Cohen, 2018 [42]	Weekly phone calls/emails from the study dietitian to review food records and discuss strategies to enhance participants’ adherence	BHB (mmol/L) I: Sig. increase C: NS change	NR	I: *n* = 6 did not enroll due to scheduling conflicts; *n* = 6 withdrew: 1 scheduling conflicts; 1 no longer wishing to comply with dietary requirements; 3 cancer recurrence; 1 death C: *n* = 10 did not enroll due to scheduling conflicts; *n* = 6 withdrew: 3 scheduling conflicts; 2 no longer wishing to comply with dietary requirements, 1 death
Khodabakhshi, 2020 [40]	BHB every 3 weeks and dietary intake	Serum ketones > 0.5 mmol/L: 66.7% Sig. increase in serum ketones in I	None reported in both groups	I: *n* = 10 withdrew after beginning assigned diet (2 nausea and hypoglycemia; 3 weakness and hunger; 1 refusal to participate; 2 unable to stick to diet; 2 lack of energy and oiliness of the diet) C: *n* = 3 patients withdrew before beginning assigned diet; *n* = 7 withdrew after beginning assigned diet (5 frequent blood sampling; 1 surgery; 1 diabetes)
Martin-McGill, 2020 [39]	Assessment of diet adherence: food diaries Assessment of ketosis: urinary ketones and blood ketones (at home)	Blood ketones: ≥4 mmol/L During the first 6 weeks: MCTKD: 79.7%; MKD: 79.3%	Hypokalemia (*n* = 2), hypernatremia (*n* = 1), hypocalcemia (*n* = 1), partial seizure (*n* = 1), post-operative wound infection (*n* = 1) seizure (*n* = 1), back pain (*n* = 1) [none related to the dietary intervention] **Gastrointestinal side effects**: First 6 weeks: MCT KD group: diarrhea (*n* = 1), nausea (*n* = 1), vomiting (*n* = 1), dyspepsia (*n* = 1); MKD group: vomiting (*n* = 1) and a dry mouth (*n* = 1) At month 6: MCTKD: diarrhea, dyspepsia, constipation (*n* = 1); MKD: constipation (*n* = 1)	MCTKD: 6 randomized: 1 withdrew prior to commencing (changed mind); 5 commenced; 2 withdrew (1 dietary burden; 1 recruited to another trial); 3 completed 12 weeks; 1 withdrew (GI intolerance); 2 completed 12 months MKD: 6 randomized: 1 withdrew prior to commencing (non-related SAE); 5 commenced; 4 withdrew (2 dietary burden; 1 tumor progression; 1 nausea); 1 completed 12 weeks; 1 completed 12 months
Neurological disorders				
Lee, 2020 [43]	Plasma BHB	Plasma BHB: ≥0.50 mmol/L Sig. higher BHB in KD than MPD and C	None reported	*n* = 1 in KD not analyzed because of large amount of missing data
Philips, 2021 [41]	Assessment of diet adherence: 3-day (2 weekdays, 1 weekend day) food record Assessment of ketosis: Bedtime ketone monitoring	Serum BHB ≥ 0.6 mmol/L 85.7% of patients who completed KD achieved sustained physiological ketosis	I: Increased irritability: 19%; Increased fatigue: 23%; Sugar craving: 8%; Insomnia: 4%; Muscle cramp: 12%; Constipation: 4%; Feeling light headed: 15%; Increased back pain: 4%; Excessive hunger: 8%; Excessive thirst: 4%; Diarrhea: 4%; Palpitations: 4% C: Increased irritability: 35%; Increased fatigue: 27%; Sugar craving: 23%; Insomnia: 19%; Muscle cramp: 4%; Constipation: 15%; Feeling light headed: 12%; Increased back pain: 12%; Nausea: 8%; Headache: 12%; Heart burn: 8%; Palpitations: 4%; Urinary calculus: 4%; Psychotic episode: 4%	Phase 1 I: *n* = 13; 2 withdrew (1 declined to remove daily sugar; 1 excess coconut oil and diarrhea); 11 completers C: *n* = 13; 13 completers Phase 2 I: *n* = 13; 10 completers; 3 withdrew (1 declined to remove daily sugar; 1 declined to remove daily beer; 1 declined most of the food) C: *n* = 13; 13 completers
Obesity and T2DM				
Brinkworth, 2016 [37]	Good compliance in both groups to prescribed diets throughout the study assessed by dietary intake	Plasma BHB increased more with I after 4 weeks and remained higher over 52 weeks than C (Sig.)	Musculoskeletal ailments: I: *n* = 8; C: *n* = 13 [Associated with exercise training: I: *n* = 6; C: *n* = 8]; Gastrointestinal disorders (constipation and diverticulitis): I: *n* = 2; C: *n* = 1, Esophageal ulcers with Helicobacter pylori infection: C: *n* = 1; Non-hospitalized hypoglycemia incident: I: *n* = 1; Hospitalization for arrhythmia with suspected heart failure: C: *n* = 1; Prostate cancer and melanoma: I: *n* = 1; C: *n* = 1; Non-study related workplace injuries: I: *n* = 3; C: *n* = 1; Hospitalization for pneumonia: I: *n* = 1; Malignant hyperthermia: I: *n* = 1; Anaphylactic reaction to the influenza vaccine: C: *n* = 1; Motor vehicle accident: C: *n* = 1	I: *n* = 17 (6 lost to follow-up; 4 time constraints; 3 work commitments; 2 unable to comply with diet; 2 personal reasons; 1 health issue external to study) C: *n* = 21 (4 lost to follow-up; 1 time constraints; 3 work commitments; 5 unable to comply with diet; 5 personal reasons; 3 health issue external to study)
Durrer, 2021 [38]	I: non-adherence: 2.12% Assessment of food intake: fasting blood sample and a 3-day diet	NR	I: *n* = 4: mild hypoglycemic events (*n* = 2 when participants were reluctant to reduce insulin dosages by the recommended amount; resolved with recommended medication); Hypoglycemic symptoms (*n* = 1 might be due to waiting too long between meals; resolved after solving this issue); Cardiac event (*n* = 1 occurred 3 weeks into the study; deemed not related to I by data and safety monitoring board) C: *n* = 0	Drop-out prior to commencing the trial: I: *n* = 4 (1 ineligible; 3 lost contact) C: *n* = 15 (2 ineligible; 1 moved away; 12 lost contact) Attrition I: *n* = 16 (2 family issues; 2 could not adhere; 2 unrelated health issues; 1 travel; 9 lost contact) C: *n* = 15 (15 lost contact)
Knee Osteoarthritis				
Strath, 2020 [44]	Adherence verbally confirmed; food journals assessed by a dietician and the study administrator at each visit	Not measured	NR	LFD: 1 lost to nonadherence C: 2 failed to complete the study

BHB: B-Hydroxybutyrate; C: Control; CHO: Carbohydrate; CI: Confidence interval; EORTC QLQ-C30: European Organization for Research and Treatment of Cancer current core; GHS: Global Health Status; I: Intervention; KD: Ketogenic diet; LCD: Low-carbohydrate diet; LFD: Low at diet; MCS: Mental component summary; MCT: Medium chain triglycerides; MCTKD: Medium chain triglyceride ketogenic diet; MKD: Modified ketogenic diet; MPD: Modified Paleolithic diet; MUFA: Monounsaturated fatty acids; NR: Not reported; NS: Not significant; PCS: Physical component summary; PUFA: Polyunsaturated fatty acids; QOL: Quality of life; SD: Standard deviation; SFA: Saturated fatty acids; Sig: Significant; T2DM: Type 2 diabetes mellitus. * indicates interaction.

### 3.5. Dietary Compliance with the Ketogenic Diet

As Table 2b shows, the results of dietary compliance with the KD were reported only in three studies [37,38,40]. Whenever investigated, good dietary compliance was reported [37,38,40], reaching 98% in the study by Durrer et al. [38], and 96% in the study by Khodabakhshi et al. [40]. Interestingly, in the latter study [40], only 66.7% of the group had serum ketones above 0.5 mmol/L at 12 weeks.

### 3.6. Adverse Events

Adverse events were not investigated in the studies by Cohen et al. [42] and Strath et al. [44]. In the studies conducted by Lee et al. [43] and Khodabakhshi et al. [40], no adverse events of the KD were noted. In the study by Augustus et al. [36], side-effects were mostly noted during the first two to six weeks, relating to the keto-induction phase, and they consisted mainly of feelings of fatigue and dizziness. In the studies by Brinkworth et al. [37] and Philips et al. [41], there were no marked differences in the adverse events between patients receiving KD and those receiving the comparison diet. Details about the specific side effects are reported in Table 2b.

## 4. Discussion

This systematic review explored the effect of KD on QOL among adults with chronic disease. Overall, we could not find conclusive evidence about the effectiveness of the different forms of the KD in improving QOL in this patient population. This finding is limited by the divergent dietary regimens used for prescribing the KD, the heterogeneity of the subjects in the studies, and chronic diseases assessed as well as the methodological variations between studies such as a comparison, and the assessment methods of ketosis and QOL.

All included publications were recent (2016–2021), reflecting increased attention to KD in the scientific literature for health-related outcomes beyond those related to the management of epilepsy [45]. The KD has been advocated for improving the QOL of healthy and ill people due to several biologically plausible mechanisms. One suggested theory is the effect of ketone bodies, specifically B-hydroxybutyrate, in inducing mild euphoria [46]. Qualitative findings from the study by Martin-McGill et al. [39] illustrate this, with patients reported “*experiencing a fantastic quality of life*” and describing the diet as offering *a sense of control* whilst receiving their tumor treatment”. BHB has shared actions with gamma-hydroxybutyrate (GHB) on the brain. GHB is a catabolite in the brain of gamma-aminobutyrate (GABA), thus lowering cerebral energy requirements and playing a neuroprotective role [47]. Another plausible theory could be the weight-loss effect of the KD. Weight loss is suggested to improve health-related QOL; this relationship is consistent with considerable loss such as the case after bariatric surgery [48]. Furthermore, the potential positive effect of KD on QOL could be attributed to its anti-inflammatory effect. It is hypothesized that excess dietary CHO contribute to oxidative stress, pain, and inflammation [44]. Chronic inflammation significantly predicts lower QOL in emotional and relational measures [49,50].

Specifically, in cancer patients, the effects of the KD on QOL could be secondary to its therapeutic effects. For example, an increasing number of preclinical studies suggest the KD as a potent anticancer therapy because of its direct effects on tumor growth, which may improve the overall health status of patients as well as their QOL [51,52]. In patients with neurological disorders such as Alzheimer’s disease, although the literature is scarce and lacks scientific rigor, KD seems to improve the cognitive symptomatology of these diseases, hence its speculated effect on QOL in this patient population [53]. The possible neuroprotective effects of the KD could potentially reside in its beneficial effect on reducing accumulation of amyloid plaques, protecting against amyloid-beta toxicity, and through the modification of the neuronal network activity, although precise mechanisms remain unknown. Furthermore, BHB has cellular signaling functions [54] that broadly link the outside environment to cellular function through epigenetic gene regulation, with potential implications on human aging [55]. Finally, the KD diet is reportedly associated with improvements in food cravings, increased levels of physical activity, sleep, and sexual function, and decreased medication need [45,56]. These effects may be of utmost interest to type 2 diabetic patients.

The scientific literature highlights the lack of a standardized definition of the KD. Commonly, this diet is characterized by a reduction in CHO below 10% of daily energy intake or typically 30 to 50 g of CHO per day, and a relative increase in fat intake, with a fat-to-CHO and protein intake ratio of 3:1 to 4:1 [57]. The included studies in this systematic review varied in their definition of the KD, the level of CHO restriction as well as protein and fat intakes, in addition to the inclusion of MCT as a source of fat. Furthermore, the use of KD and induction of ketosis were not explicit in some included studies, nevertheless, the features of the interventional diet were in accordance with that of a KD.

There was also a variation in the definition of physiological ketosis with different cut-off values used in different studies as well as the assessment of ketosis, varying between blood and urinary ketones. Specifically, regarding the latter method, urinary ketones were used to monitor the diet in numerous studies [36,39,42]. Urinary ketones may not be robust markers of compliance, as they can be affected by hydration status, some medications or substances such as vitamin C. Furthermore, the reading is an average of urine ketone levels since the last void. Finally, some kits only measure acetoacetic levels and others have a short shelf-life [56]. Of interest, apparently low urinary ketones may discourage patients when they are adhering to the diet robustly [39]; this is to be considered for future RCTs investigating the KD. In addition, only the two studies conducted by Augustus et al. [36] and Cohen et al. [42] reported on the relationship between ketone bodies and QOL. The results of the two studies were contradictory: while the first reported an inverse association between urinary ketones and QOL, the latter reported no association between physical nor mental health and serum BHB, hence a conclusion on this relationship cannot be drawn.

Importantly, participant blinding was impossible in all trials. It is thus likely that improvements noted with the KD may be biased by the increased attention given to patients through consultations, education, and follow-up, and by the fact that patients had the sense that they were actively participating in treatment to enhance their disease condition [36]. These factors were not measured in the trials, hence cannot be controlled with the analysis. Furthermore, remarkable adherence was noted in this systematic review. This finding should be interpreted with caution since only three studies (i.e., Brinkworth et al. [37], Durrer et al. [38], and Khodabakhshi et al. [40]) reported the results of the dietary compliance, and in these studies, compliance was self-reported, which may be subject to recall bias, error and inaccuracy, or social desirability bias [58]. Low adherence to the KD in adult patients with cancer was reported in a recent systematic review (49%) [25]. Adherence to the KD requires drastic changes, which could hinder long-term compliance [59]. Indeed, Augustus et al. [36] presented the case that the KD may not be offered to all patients and that being unable to adhere to the KD or struggling to be adapted to it may adversely affect the QOL and mental health of patients.

Interestingly, in the majority of included studies, adverse events did not greatly differ between the KD and its comparison. Furthermore, commonly reported side effects with the KD included fatigue, dizziness, and gastrointestinal symptoms. These effects, mainly reported in the first weeks of adopting the KD, are attributed to the keto-induction period, which varies greatly inter-individual [60,61]. Specifically, Augustus et al. [36] reported a small but distinct gender-based difference in this period, whereby men adapted faster to the KD as well as an age-based difference, with younger individuals adapting faster than their older counterparts. The keto-induction period involves a course of immediate side-effects known as the keto-flu, which coincide with a high level of ketone bodies. These effects steadily attenuate over time with keto-adaptation (i.e., organ homeostasis to using ketones as the primary source of energy [60]).

### Strengths and Limitations

This was the first study to compile evidence from RCTs on the effect of KD diet on QOL in adults with chronic diseases. We adopted a sensitive search strategy, followed the recommended methods for applying the search, selection of studies, data extraction, and quality assessment as well as best-practices for the reporting of the review [30,31]. Moreover, when studies were lacking information, original references were retrieved for additional data on design and results. Finally, the risk of bias of the majority of included studies was low, except for the allocation concealment and blinding of participants and personnel.

On the other hand, the review is limited by the heterogeneity of included studies, which complicates the comparisons and the interpretation of the findings, specifically regarding assessed populations, assessment methods, and study outcomes. Additionally, some studies lacked information crucial for the interpretation of the results such as baseline QOL values, adverse events, assessment of ketosis, and proportion of participants with sustained physiological ketosis throughout the trial. Having such information would have helped us interpret the findings in a better manner. Moreover, the search, despite being highly broad and sensitive, might have missed some relevant studies. This limitation is common to systematic reviews. Other limitations related to being unable to access one potentially eligible study for full text screening, and excluding additional two studies in this phase due to lack of information on randomization and unavailability of outcome data in the published article. We tried to contact the respective authors, without an answer. Finally, given the heterogeneity of the studies included, meta-analyzing their results was impossible.

## 5. Conclusions

In conclusion, the evidence from RCTs investigating the effect of KD on QOL in adults with chronic disease is inconclusive. The promising effect noted in some included studies, and the low rates of adverse events and side effects encourage future investigations in this regard. Hence, additional high-quality, powered trials with long enough follow-up periods, are warranted to elucidate the effect of the KD on QOL in adults with chronic disease and explore the optimal diet composition and timing of initiation for optimal outcomes.

## Supplementary Materials

The following are available online at https://www.mdpi.com/article/10.3390/nu13124463/s1, Table S1: Characteristics of included studies., Table S2: **a**. Results of included studies; **b.** Results of included studies.
