# Supplementary material for: Effect of Ketogenic Diet on Quality of Life in Adults with Chronic Disease: A Systematic Review of Randomized Controlled Trials
Source: Nutrients. 2021 Dec 14;13(12):4463. doi: 10.3390/nu13124463 (PMC8708168; doi:10.3390/nu13124463)
Supplement: Supplementary file 1 [file nutrients-13-04463-s001.zip › nutrients-1503746-supplementary.pdf]

Table S1. Characteristics of included studies.

First Author, Year	Country	Study population & Type of chronic disease	Age (Range/Mean±SD)	%Male	Duration	Intervention: Features of KD	Control	Isocaloric diets (arms)	Co-intervention	Assessment of Ketosis	Assessment of QOL
Cancer											
Augustus, 2021 [39]	Trinidad and Tobado (Trinidad)	Stage 2 and 3 cancer patients (breast (65%), prostate (44%), colon/rectal (17%), cervical (13%), and lung (6%)) diagnosed with cancer >1 year, >18 years, currently receiving chemotherapy or radiation treatment, nonvegetarian, consuming a CHO-based diet (>40%), recruited from at an outpatient private clinic<br>I: n = 20; 16 completed the trial<br>C: n = 20; 20 completed the trial	mean(SD):<br>I: 49.80 ± 6.72<br>C: 51.80 ± 4.18	NR	16 weeks	MKD: 7-day cyclic altered KD menu developed to avoid repetition and lack of interest in the meals (≈10% CHO (50g), 15% Protein (75g), 75% Fat (167g) providing 2,000 Kcal); main source of Fat: MCT	Standard traditional diet: usual diet with minor alterations being made to ensure that daily energy and nutrient requirements are met	Not specified by study protocol	None	Urinary ketones: dip stick test and urine analyzer	EORTC QLQ-C30 questionnaire: comprising 9 multi-item scales (5 functional scales: cognitive, physical, role, emotional, social; 3 scales to evaluate symptoms: pain, fatigue, nausea, vomiting; an overall global health and QOL scale), and several single item symptom measures
Cohen, 2018 [36]	Birmingham (USA)	Women with ovarian or endometrial cancer, BMI ≥18.5 kg/m2, ≥19 years, without medical condition affecting body weight and not attempting diet modification, recruited from a gynecologic oncology clinic and treatment centers<br><br>I: n = 37; 25 completed the trial<br>C: n = 36; 20 completed the trial	I: 61.5 ± 8.5<br>C: 58.6 ± 11.7	0%	12 weeks	KD: 5% CHO (≤20g); 25% Protein (≤100g); 70% Fat (≤125g)	ACS diet: based on recommendations from the American Cancer Society and the Academy of Nutrition and Dietetics including increased intake of fiber-rich fruits, vegetables, whole grains, as well as lean meats and small amounts of healthful fats	Neither group was instructed to alter total energy intake	None	Serum BHB: SIRRUS analyzer<br>Urinary ketones: strips	SF-12: a score from 0 to 100, with higher scores indicative of better functional status (PCS and MCS)
Khodabakhshi, 2020 [43]	Tehran (Iran)	80 women with locally advanced or metastatic breast cancer receiving chemotherapy for at least 12 weeks<br>I: n=40; 30 completed the trial (83.3% locally advanced; 16.7% metastatic)<br>C: n=40; 30 completed the trial (63.3% locally advanced; 36.7% metastatic)	Range: 18-70<br>I: 44.8 ± 8.4<br>C: 45.2 ± 15.0	0%	12 weeks	6% CHO, 19% Protein, 20% MCT, 55% Fat	55% CHO, 15% Protein, 30% Fat	Both diets calculated to be eucaloric using the Mifflin-St. Jeor formula	None	Blood BHB: home kit	EORTC QLQ-C30 and EORTC QLQ-BR23 questionnaires developed by the European Organization for Research and Treatment of Cancer
Martin-McGill, 2020 [42]	United Kingdom	12 patients (≥16 years) diagnosed with glioblastoma within the last 4 months (WHO grade IV) planning to go temozolomide chemotherapy and radiotherapy<br>MKD: n= 6; 1 completed 12 weeks; 1 completed 12 months	Median age: 57y<br>Range: 44-66y	66.60%	12 weeks<br>12 months	I1: MKD: 5% CHO, 80% Fat, 15% Protein<br>I2: MCTKD: 10% CHO, 75% Fat (30% from MCT nutrition product), 15% Protein	None	Not specified by study protocol	None	Urinary ketones: dip stick test<br>Blood ketones: home kit	EORTC QLQ C30 questionnaire<br>BN20 questionnaire

		MCTKD: n= 6; 3 completed 12 weeks; 2 completed 12 months									
Neurological disorders											
Lee, 2020 [37]	Iowa (USA)	15 patients with relapsing remitting multiple sclerosis or progressive relapsing-remitting multiple sclerosis with an expanded disability status score≥ 4.5<br>KD: n= 5; 4 analyzed (1: insufficient data)<br>MPD: n= 6<br>Usual diet: n= 4	Total: Range: 36-63<br>Mean: 51.9 ± 9.5<br><br>KD: 51.8 ± 11.8<br>MPD: 50.3 ± 9.5<br>C: 54.5 ± 11.8	50%	12 weeks	MCT-based KD: ketogenic version of the modified Paleolithic diet with additional requirements (no starchy vegetables or fruits; reduce vegetable consumption to 6 servings daily; increase fat intake with supplemental MCTs to achieve a daily goal of 70% of total Kcal from fat)	MPD: Modified Paleolithic diet: 9 daily recommended servings of vegetables comprised of leafy green vegetables, sulfur rich vegetables, and deeply colored fruits and vegetables; encourages plant and animal protein, seaweed, nutritional yeast, nondairy milks; excludes gluten-containing grains, eggs, casein<br><br>C: Usual diet	Not specified by study protocol	Pre-study vitamins, supplements, and/or medications	Plasma BHB: NR	Multiple Sclerosis Quality of Life-54: validated 54-item measure of physical- and mental health-related QOL, composed of 12 subscales, higher scores indicating better QOL
Philips, 2021 [44]	Hamilton (New Zealand)	26 patients with Alzheimer diseases (dementia severity rating scale score <19), BMI> 18.5 Kg/m2, and a cohabiting trial partner willing to partake<br>**Phase 1**<br>KD: n=13; 11 completed the phase<br>Usual diet: n=13; 13 completed the phase<br>**Phase 2**<br>KD: n=13; 10 completed the phase<br>Usual diet: n=13; 13 completed the phase	**Total:**<br>Range: 57-79<br>Mean: 69.8 ± 6.0<br>**KD > Usual diet**<br>Range: 57-77<br>Mean: 68.0 ± 5.4<br>**Usual diet > KD**<br>Range: 61-79<br>Mean: 71.7 ± 6.2	Total: 62%<br>KD > Usual diet: 77%<br>Usual diet > KD: 46%	12 weeks: I or C<br>10 weeks: washout	58% Fat (26% SFA, 32% non-saturated), 29% Protein, 7% Fiber, 6% net CHO by weight	Usual diet: optional low-fat recipes in accordance with New Zealand healthy-eating guidelines; 11% Fat (3% SFA, 8% non-saturated), 19% Protein, 8% Fiber, 62% net CHO by weight	Not specified by study protocol	Daily multivitamin	Serum BHB: ketone blood monitor	QOL-AD administered to the trial partner: 13 items, scores between 13-52, with higher numbers indicating better QOL
Obesity and T2DM											
Brinkworth, 2016 [40]	Adelaide (Australia)	Adults with T2DM (HbA1c ≥7.0% or taking a diabetes medication), overweight and obese (BMI: 26-45 kg/m2), recruited via a public advertisement<br>I: n = 58; 41 completed the trial;	Range: 35-68<br>I (n = 58): 58 ± 72<br>C (n = 57): 58 ± 7	I: 64%<br>C: 51%	12 months	Energy-restricted very-low CHO, high-fat diet: 14% CHO (<50g); 28% Protein, 58% Fat (35% MUFA, 13% PUFA, <10% SFA)	Energy-restricted high-CHO, low-fat diet: 53% CHO; 17% Protein; <30% Fat (15% MUFA, 9% PUFA, <10% SFA)	Isocaloric I and C diets designed to be moderately energy	Multicomponent 60-minute, moderate-intensity, exercise program delivered and	Plasma BHB: D-3 Hydroxybutyrate kit	Diabetes-39 Questionnaire: assesses 5 dimensions of diabetes-related QOL: anxiety/worry (4 items); social burden (5 items); diabetes control (12

		71% retention<br>C: n = 57; 37 completed the trial; 65% retention						restricted (~30% restriction; 500-1000 Kcal/day deficit)	supervised by exercise professionals on three nonconsecutive days/week		items); sexual functioning (3 items); energy and mobility (15 items) [each item is on a Likert scale: 1 (not affected at all) to 7 (extremely affected)]
Durrer, 2021 [41]	Southern British Columbia (Canada)	Adults with T2DM diagnosed by a physician, using at least one glucose-lowering medication, obese (BMI ≥30 kgm2), recruited from community pharmacies<br><br>I: n = 98; n=78 completed the trial (98 ITT)<br>C: n = 90; n = 60 completed the trial (90 ITT)	I: 58 ± 11<br>C: 59 ± 8	I: 44%<br>C: 43%	12 weeks	Low-CHO energy-restricted commercial weight loss plan supplemented with whole foods (<50g CHO; 35–45 g Fat, 110–120 g Protein; 850-1100 Kcal)	Treatment-as-usual delivered by community pharmacists: information pamphlets on diet and lifestyle conforming with 2013 Diabetes Canada Clinical Practice Guidelines	Not specified by study protocol	None	Capillary blood ketones: NR	SF-20
Knee Osteoarthritis											
Strath, 2020 [38]	Birmingham (USA)	21 adults with medically diagnosed knee osteoarthritis<br>LCD: n= 8<br>LFD: n=6<br>C: n=7	Range: 65-75<br>LCD: mean 71.00 ± 3.12<br>LFD: mean 72.33 ± 1.97<br>C: mean 68.71 ± 7.11	Randomized:<br>LCD: 60%<br>LFD: 75%<br>C: 80%<br><br>Completers:<br>LCD: 60%<br>LFD: 100%<br>C: 75%	12 weeks	Kcal: unlimited; Fat: unlimited; CHO: 20g; Proteins: 100g	LFD: Kcal: 800-1200; Fat: 50-67g; CHO: unlimited; Proteins: 100g<br>C: Kcal: unlimited; Fat: unlimited; CHO: unlimited; Proteins: unlimited	No	None	Not measured	Knee Injury and Osteoarthritis Outcome Score quality of life questionnaire

ACS: American Cancer Society; BHB: B-hydroxybutyrate; BMI: Body mass index; C: Control; CHO: Carbohydrate; EORTC QLQ-C30: European Organization for Research and Treatment of Cancer current core; GHS: Global Health Status; HbA1c: Glycated hemoglobin; I: Intervention; ITT: Intention to treat; KD: Ketogenic diet; LCD: Low-carbohydrate diet; LFD: Low fat diet; MCS: Mental component summary; MCT: Medium chain triglycerides; MCTKD: Medium chain triglyceride ketogenic diet; MKD: Modified ketogenic diet; MPD: Modified Paleolithic diet; MUFA: Monounsaturated fatty acids; NR: Not reported; PCS: Physical component summary; PUFA: Polyunsaturated fatty acids; QOL: Quality of life; QOL-AD: Quality of Life in Alzheimer's Disease; SD: Standard deviation; SF-12: Medical Outcomes Study Short Form-12 Health Survey; SF-20: Medical Outcomes Study Short Form-20 Health Survey; SFA: Saturated fatty acids; T2DM: Type 2 diabetes mellitus; WHO: World Health Organization.

Table S2. a. Results of included studies.

First Author, Year	Baseline QOL	Endline QOL	Conclusion
Cancer			
Augustus, 2021 [39]	I: 64.85 ± 7.9<br>C: 61.05 ± 13.81 (NS)	I: 36.850 ± 7.576<br>C: 60.450 ± 14.898 (Sig. difference over time; effect size: 0.268, medium)<br>Regression: inverse association between urinary ketones and QOL (b=-3.175, 95% CI=-5.723, –0.626); among the predictors: patients being in I group (b=–5.241, 95% CI= –10.373, –0.109)	Keto-adapted patients on a MKD had an improvement in self-reported QOL over time<br>KD may improve QOL of cancer patients (not inclusive of advanced stage cancer) compared with patients on a standard traditional diet
Cohen, 2018 [36]	NR	PCS: I: 45; C: 40 (Sig)<br>MCS: I: 53; C: 53 (NS)<br>[Approximate numbers estimate from a figure in the article]<br>NS between-group difference in PCS after adjusting for baseline values, chemotherapy status, and fat<br>Sig. within-group increase in PCS in I group (11%); NS in C group<br>NS difference in the MCS between or within the diet groups<br>NS association between the PCS or MCS and serum BHB	Sig. between-group difference in adjusted PCS, NS between-group differences in MCS<br>In women with ovarian or endometrial cancer, a KD does not negatively affect quality of life and may improve physical function
Khodabakhshi, 2020 [43]	**EORTC QLQ-C30 Functioning (mean ± SD)**<br>**[Higher values indicate higher level of functioning and QOL]**<br>Physical functioning: I: 89 ± 11; C: 76 ± 20 (Sig. difference between groups)<br>Role functioning: I: 86 ± 16; C: 79 ± 28<br>Cognitive functioning: I: 85 ± 16; C:71 ± 28 (Sig. difference between groups)<br>Emotional functioning: I: 67 ± 21 ; C: 66 ± 21<br>Social functioning: I: 94 ± 17; C: 93 ± 17<br>Global quality of life: I:68 ± 16; C: 65 ± 16<br><br>**EORTC QLQ-C30 Symptoms (median (quartiles 25, 75))**<br>**[Higher values indicate a higher grade of symptoms]**<br>Fatigue: I: 22(8–33); C: 33(11–44) (NS between groups)<br>Nausea & vomiting: I: 0(0–0); C: 0 (0–20) (NS between groups)<br>Pain: I: 16(0–50); C: 16(0–33) (NS between groups)<br>Reduction in appetite: I: 0(0–8); C: 0(0–33) (NS between groups)<br>Sleep difficulties: I: 16(0–33); C: 0(0–66) (NS between groups)<br>Dyspnea: I: 0(0–33); C: 0(0–33) (NS between groups)<br>Constipation: I: 0 (0–0); C: 0 (0–33) (NS between groups)<br>Diarrhea: I: 0 (0–0); C: 0 (0–0) (NS between groups)<br>Financial concerns: I: 0 (0–0); C: 0 (0–0) (NS between groups)<br><br>**EORTC QLQBR23 Functioning (median (quartiles 25, 75))**<br>**[Higher values indicate higher level of functioning and QOL]**<br>Future perspective: I: 66 (33–100); C: 66 (33–66) (NS between groups)<br><br>**EORTC QLQBR23 Symptoms (median (quartiles 25, 75))**<br>**[Higher values indicate a higher grade of symptoms]**<br>Arms: I: 11 (0–36); C: 11 (0–22) (NS between groups)<br>Breast: I: 8 (0–33); C: 8 (0–25) (NS between groups)<br>Systemic therapy side effect: I: 9 (4–17); C: 14 (4–23) (NS between	**EORTC QLQ-C30 Functioning (mean ± SD)**<br>**[Higher values indicate higher level of functioning and QOL]**<br>Physical functioning: I: 78 ± 19; C: 68 ± 20 (NS between groups; Sig. diff in I)<br>Role functioning: I: 75 ± 25; C: 66 ± 29 (NS between groups; Sig. diff in C)<br>Cognitive functioning: I: 75 ± 19; C: 72 ± 21 (NS between groups; Sig. diff in I)<br>Emotional functioning: I: 62 ± 23; C: 60 ± 21 (NS between groups)<br>Social functioning: I: 91 ± 17; C: 87 ± 17 (NS between groups; Sig. diff in C)<br>Global quality of life: I: 70 ± 20; C: 62 ± 20 (NS between groups)<br><br>**EORTC QLQ-C30 Symptoms (median (quartiles 25, 75))**<br>**[Higher values indicate a higher grade of symptoms]**<br>Fatigue: I: 33(19–55); C: 33(33–55) (NS between groups; Sig. diff in I; Sig. diff in C)<br>Nausea & Vomiting: I: 33(19–55); C: 33(33–55) (NS between groups; Sig. diff in I; Sig. diff in C)<br>Pain: I: 16(0–50); C: 33(16–50) (NS between groups)<br>Reduction in appetite: I: 33(0–33); C: 16(0–33) (NS between groups; Sig. diff in I)<br>Sleep difficulties: I: 0(0–41); C: 33(0–50) (NS between groups)<br>Dyspnea: I: 33(0–41); C: 16(0–33) (NS between groups)<br>Constipation: I: 0 (0–33); C: 0 (0–33) (NS between groups)<br>Diarrhea: I: 0 (0–33); C: 0 (0–0) (NS between groups)<br>Financial concerns: I: 0 (0–33); C: 33 (0–58) (NS between groups; Sig. diff in C)<br><br>**EORTC QLQBR23 Functioning (median (quartiles 25, 75))**<br>**[Higher values indicate higher level of functioning and QOL]**<br>Future perspective: I: 66 (33–66); C: 33 (33–100) (NS between groups)<br><br>**EORTC QLQBR23 Symptoms (median (quartiles 25, 75))**<br>**[Higher values indicate a higher grade of symptoms]**<br>Arm: I: 11 (0–36); C: 22 (0–33) (NS between groups)	After adjusting baseline values and chemotherapy status, NS differences in all domains of QOL between I and C<br>KD diet combined to chemotherapy in patients with breast cancer does not bring additional benefit about QOL at 12 weeks

	groups)<br>Concerns over hair loss: I: 0; C: 0 (NS between groups)	Breast: I: 8 (0–10); C: 8 (0–16) (NS between groups; Sig. diff in I)<br>Systemic therapy side effect: I: 42 (20–52); C: 42 (33–52) (NS between groups; Sig. diff in I; Sig. diff in C)<br>Concerns over hair loss: I: 66 (33–100); C: 33 (33–100) (NS between groups; Sig. diff in I; Sig. diff in C)	
Martin-McGill, 2020 [42]	Little difference between GHS between patients who withdrew and those who were retained, in either dietary group:<br>MCTKD: withdrew (n=3): 72.2 ± 20.7; retained (n=3): 75 ± 6.8<br>MKD: withdrew (n=5): 70 ± 13.8; retained (n=1): 80 ± 0	**Week 6:** GHS of those who withdrew fell below the brain cancer reference value in both the MCTKD and MKD groups (withdrew: MCTKD 41.7±0 [n = 1]; MKD 50 ± 0 [n = 2])<br>**Week 6 onward:** GHS improved for the patient following MKD and reduced for patients following MCTKD. In both groups, GHS remained above the brain cancer reference value: retained at month 12: MCTKD: 66.7±8.4 [n=2]; 100±0 [n=1]	GHS was below the brain cancer reference value for patients at week 6 of the study<br>For retained patients at 12 months, GHS reduced within the MCTKD group and improved in the MKD group
		Neurological disorders	
Lee, 2020 [37]	**Mental Health**<br>KD: 61.5 ±25.5<br>MPD: 53.7±23.3<br>C: 66.3± 8.2<br><br>**Physical Health**<br>KD: 40.4 ±6.2<br>MPD: 32.8±10.8 (sig. lower than usual diet)<br>C: 46.4 ±11.0	**Mental Health (NS difference between groups)**<br>KD: ≈67 (data from graph)<br>MPD: 67.0± 24.7<br>C: 54.1± 21.1<br><br>**Physical Health (NS difference between groups)**<br>KD: ≈40 (data from graph)<br>MPD: 47 ±18<br>C: 40.7 ±14.4	NS differences in mental health and physical health QOL scores among groups<br>Suggested clinically sig. improvements in mental health and physical health QOL with Modified Paleolithic diet (change >5)<br>Suggested clinically sig. decline in mental health and physical health QOL with usual diet
Philips, 2021 [44]	**Phase 1:** KD > Usual diet: 33.8 ± 5.6; Usual diet > KD: 33.9 ± 5.8<br>**Phase 2:** KD > Usual diet: 34.1 ± 8.5; Usual diet > KD: 31.8 ± 7.3<br>**Total:** KD > Usual diet: 32.8 ± 6.4; Usual diet > KD: 34.0 ± 7.1<br><br>**Patients who remained on protocol for both treatment periods and achieved sustained physiological ketosis in first treatment period:** KD > Usual diet: 35.4 ± 5.0; Usual diet > KD: 32.1 ± 5.8<br>**Patients who remained on protocol for both treatment periods and achieved sustained physiological ketosis in second treatment period:** KD > Usual diet: 36.9 ± 7.5; Usual diet > KD: 30.3 ± 8.2<br>**Total:** KD > Usual diet: 33.1 ± 6.9; Usual diet > KD: 34.8 ± 7.1	**Phase 1:** KD > Usual diet: +2.86 ± 4.64; Usual diet > KD: −1.15 ± 5.41<br>**Phase 2:** KD > Usual diet: +0.31 ± 3.68; Usual diet > KD: +3.03 ± 7.52<br>**Total:** KD > Usual diet: +2.95 ± 6.12; Usual diet > KD: −0.42 ± 4.60<br>Sig. treatment effect (+3.37 ± 6.86)<br>**Patients who remained on protocol for both treatment periods and achieved sustained physiological ketosis in first treatment period:** KD > Usual diet: +3.50 ± 4.38; Usual diet > KD: −0.63 ± 6.93<br>**Patients who remained on protocol for both treatment periods and achieved sustained physiological ketosis in first treatment period:** KD > Usual diet: -0.70 ± 3.59; Usual diet > KD: +3.75 ± 9.56<br>**Total:** KD > Usual diet: +3.61 ± 6.91; Usual diet > KD: −0.67 ± 5.16<br>Sig. treatment effect (+4.28 ± 7.27)	Patients on KD had improved QOL as compared to those on usual diet<br>High rates of retention and adherence are achievable in applying a 12-week MKD to patients with Alzheimer's disease and adverse effects are mild
		Obesity and T2DM	
Brinkworth, 2016 [40]	Anxiety and Worry<br>I: 31.8 ± 2.9<br>C: 25.9 ± 2.9<br>Social Burden<br>I: 9.3 ± 1.7<br>C: 7.7 ± 1.7<br>Sexual Functioning<br>I: 24.3 ± 4.0<br>C: 22.8 ± 4.1<br>Energy and Mobility<br>I: 18.1 ± 2.1<br>C: 17.5 ± 2.1	Anxiety and Worry<br>I: 31.5 ± 3.8<br>C: 22.1 ± 3.9<br>Social Burden<br>I: 10.8 ± 2.3<br>C: 8.1 ± 2.3<br>Sexual Functioning<br>I: 19.6 ± 3.5<br>C: 11.9 ± 3.5<br>Energy and Mobility<br>I: 19.3 ± 2.3<br>C: 12.5 ± 2.3	In overweight and obese adults with T2DM, both high and low CHO diets achieved comparable improvements in QOL
Durrer, 2021 [41]	Physical Functioning<br>I: 70.1 ± 28.8	Adjusted means (*a precise p-value could not be obtained)<br>Physical Functioning: Treatment effect: 0.7 (−7.7, 9.9)*	In obese patients with T2DM, there sig. improvement in role functioning, mental health,

	C: 69.6 ± 28.2<br>Role Functioning<br>I: 71.9 ± 39.1<br>C: 71.7 ± 40.7<br>Social Functioning<br>I: 81.3 ± 27.9<br>C: 84.4 ± 25.4<br>Mental Health<br>I: 76.7 ± 16.6<br>C: 74.4 ± 16.3<br>Health Perceptions<br>I: 52.8 ± 24.4<br>C: 49.2 ± 23.6<br>Pain<br>I: 41.6 ± 27.0<br>C: 47.6 ± 21.3	I: 72.5<br>C: 71.8<br>Role Functioning: Treatment effect: 13.6 (2.4, 26.3)*<br>I: 88.6<br>C: 75.0<br>Social Functioning: Treatment effect: 6.1 (−2, 14.3)*<br>I: 93.8<br>C: 87.8<br>Mental Health: Treatment effect: 6.9 (1.9, 12.7)*<br>I: 83.4<br>C: 76.5<br>Health Perceptions: Treatment effect: 19.2 (13.2, 25.4) (NS)<br>I: 70.6<br>C: 51.4<br>Pain: Treatment effect: −7.5 (−17.2, −0.1)*<br>I: 28.5<br>C: 36.0	health perceptions, and pain with low-CHO energy-restricted diet compared with usual diet
Knee Osteoarthritis			
Strath, 2020 [38]	LCD: 3.28 ± 0.604<br>LFD: 2.91 ± 0.785<br>C: 3.67 ± 0.718	LCD: 2.75 ± 0.627 (sig. change from baseline to end line)<br>LFD: 2.08 ± 0.954 (NS change from baseline to end line)<br>C: 3.26 ± 0.759 (sig. change from baseline to end line)<br>NS time*diet interaction and NS differences in LCD and LFD group after post hoc analysis	In older adults with knee osteoarthritis following an LCD for 12 weeks, the QOL sig. improved. NS differences in LCD and LFD group were noted after post hoc analysis

Table S2. b. Results of included studies.

First Author, Year	Compliance	Ketosis	Adverse events/Side effects	Attrition
			Cancer	
Augustus, 2021 [39]	Three-day food diaries (2 weekdays and 1 weekend) obtained at the weeks 6 and 12 to ensure subjects were adhering ro diet	Sig. rise in urinary ketones in I group compared with C group	I: side-effects related to keto-adaptation (first 2-6 weeks; sig. reduced 6 weeks post treatment): fatigue, dizziness, reduced energy<br>C: headaches/migraines<br>Unable to determine whether reduced energy or fatigue are attributed to I or by the natural progression of the disease	I: 2% [n=4: nausea and vomiting related to I affecting subjects' palatability (n=2); inability to complete testing at all follow-up times (n=1); mortality not related to medical treatment nor I (n=1)]<br>C: 0%
Cohen, 2018 [36]	Weekly phone calls and/or e-mails from the study dietitian for the remainder of the intervention to review food records and discuss strategies to enhance participants' adherence and enjoyment of their assigned diets	BHB (mmol/L)<br>I: Week 0: 0.36 ± 0.15; Week 12: 0.91 ± 0.16 (Sig)<br>C: Week 0: 0.20 ± 0.02; Week 12: 0.25 ± 0.04	NR	I: n=6 did not enroll due to scheduling conflicts; n=6 withdrew: scheduling conflicts (n=1); no longer wishing to comply with dietary requirements (n=1); cancer recurrence n=3); death (n=1)<br>C: n=10 did not enroll due to scheduling conflicts; n=6 withdrew: scheduling conflicts (n=3); no longer wishing to comply with dietary requirements (n=2), death (n=1)
Khodabakhshi, 2020 [43]	I: BHB every 3 weeks and dietary intake at baseline and endline<br>Serum ketones >0.5 at week 12: 66.7%<br>CHO <50g: 96%; <10% Kcal from CHO: 79.2%	Serum ketones > 0.5 mmol/L: 66.7%<br>Sig. increase in serum ketones in I group (0.007 ± 0.026 to 0.923 ± 0.699 mmol/l)	None reported in both groups	I: n=10 withdrew after beginning assigned diet (2 nausea and hypoglycemia; 3 weakness and hunger; 1 refusal to participate; 2 unable to stick to diet; 2 lack of energy and oiliness of the diet)<br>C: n=3 patients withdrew before beginning assigned diet; n=7 withdrew after beginning assigned diet (5 frequent blood sampling; 1 surgery; 1 diabetes)
Martin-McGill, 2020 [42]	Assessment of diet adherence: food diaries<br>Assessment of ketosis: urinary ketones and blood ketones (monitored at home)<br>During the first 6 weeks, 79.7% of MCTKD (n=3) and 79.3% of MKD (N=3): blood ketones ≥4 mmol/L.	Blood ketones: ≥ 4mmol/L	5 adverse events: hypokalemia (n =2), hypernatremia (n = 1), hypocalcaemia (n = 1), partial seizure (n=1)<br>3 serious adverse events: post-operative wound infection (n=1) seizure (n = 1), back pain (n=1) [none related to the dietary intervention]<br>**Gastrointestinal side effects**:<br>First 6 weeks: MCT KD group : diarrhoea (n=1), nausea (n=1), vomiting (n=1), dyspepsia (n=1); MKD group: vomiting (n=1) and a dry mouth (n=1).<br>At month 6: MCTKD: diarrhoea, dyspepsia, constipation (n=1); MKD: constipation (n=1)	MCTKD: 6 randomized: 1 withdrew prior to commencing (changed mind); 5 commenced; 2 withdrew (1 dietary burden; 1 recruited to another trial); 3 completed 12 weeks; 1 withdrew (GI intolerance); 2 completed 12 months<br><br>MKD: 6 randomized: 1 withdrew prior to commencing (non-related SAE); 5 commenced; 4 withdrew (2 dietary burden; 1 tumor progression; 1 nausea); 1 completed 12 weeks; 1 completed 12 months
			Neurological disorders	
Lee, 2020 [37]	NS between-group difference in mean plasma BHB at baseline<br>At 12 weeks, KD group maintained nutritional ketosis and sig. higher BHB (0.78 ± 0.46 mmol/L; sig. higher than MPD and C)	Plasma BHB: ≥0.50 mmol/L	None reported	n=1 in KD not analyzed because of large amount of missing data
Philips, 2021 [44]	Assessment of diet adherence: 3-day (2 weekdays, 1 weekend day) food record<br>Assessment of ketosis: Bedtime ketone monitoring; 85.7% (18/21) of patients who completed KD achieved sustained physiological ketosis	Serum BHB≥ 0.6 mmol/L	**KD**<br>Increased irritability: (19%); Increased fatigue: (23%); Sugar craving: (8%); Insomnia: (4%); Muscle cramp: (12%); Constipation: (4%); Feeling light headed: (15%); Increased back pain: (4%); Excessive hunger: (8%); Excessive thirst: (4%); Diarrhea:(4%); Palpitations: (4%)<br>**Usual Diet:**<br>Increased irritability: (35%); Increased fatigue: (27%); Sugar craving:	Phase 1<br>KD: n=13; 2 withdrew (1 declined to remove daily sugar; 1 excess coconut oil and diarrhea); 11 completed the phase<br>C: n=13; 13 completed the phase<br>Phase 2<br>KD: n=13; 10 completed the phase; 3 withdrew (1 declined to remove daily sugar; 1 declined to remove daily beer; 1

			(23%); Insomnia: (19%); Muscle cramp: (4%); Constipation: (15%); Feeling light headed: (12%); Increased back pain: (12%); Nausea: (8%); Headache: (12%); Heart burn: (8%); Palpitations: (4%); Urinary calculus: (4%); Psychotic episode: (4%)	declined most of the food)<br>C: n=13; 13 completed the phase
		Obesity and T2DM		
Brinkworth, 2016 [40]	Good compliance in both groups to prescribed diets throughout the study:<br>CHO: I: 54.0-74.0 g/d (13.4–16.6% energy); C: 202.0-217.6 g/d (50.5–49.0%)<br>Protein: I: 103.1-106.1 g/d (27.0–25.6%); C: 72.1-78.5 g/d (18.9–18.4%)<br>Fat: I: 95.9-101.5 g/d (54.4–52.5%); C: 42.4-51.8 g/d (24.1-26.1%)<br>SFA: I: 9.8–11.0%; C: 7.2–8.5%<br>MUFA: I: 30.7–28.8%; C: 11.4–12.0%<br>PUFA: I: 12.4–11.1%; C: 4.1–4.2%	Plasma BHB increased more with I after 4 weeks and remained higher over 52 weeks than C (sig)	Musculoskeletal ailments: I: n = 8; C: n = 13 [Associated with exercise training: I: n = 6; C: n = 8]<br>Gastrointestinal disorders (constipation and diverticulitis): I: n = 2; C: n = 1<br>Esophageal ulcers with Helicobacter pylori infection: C: n = 1<br>Nonhospitalized hypoglycemia incident: I: n = 1<br>Hospitalization for arrhythmia with suspected heart failure: C: n = 1<br>Prostate cancer and melanoma: I: n = 1; C: n = 1<br>Nonstudy related workplace injuries: I: n = 3; C: n = 1<br>Hospitalization for pneumonia: I: n = 1<br>Malignant hyperthermia: I: n = 1<br>Anaphylactic reaction to the influenza vaccine: C: n = 1<br>Motor vehicle accident: C: n = 1	I: n=17 [Lost to follow-up (n=6); Time constraints (n=4); Work commitments (n=3); Unable to comply with diet (n=1); Personal reasons (n=2); Health issue external to study (n=1)]<br><br>C: n=21 [Lost to follow-up (n=4); Time constraints (n=1); Work commitments (n=3); Unable to comply with diet (n=5); Personal reasons (n=5); Health issue external to study (n=3)]
Durrer, 2021 [41]	I: non adherence: 2.12% (2/94)<br>Assessment of food intake: fasting blood sample and a 3-day diet	NR	I: n=4: mild hypoglycemic events (n=2 when participants were reluctant to reduce insulin dosages by the recommended amount; resolved with recommended medication); Hypoglycemic symptoms (n=1 might be due to waiting too long between meals; resolved after solving this issue); Cardiac event (n=1 occurred 3 weeks into the study; deemed not related to I by data and safety monitoring board)<br>C: n=0	Drop-out prior to commencing the trial:<br>I: n=4 (1 ineligible; 3 lost contact)<br>C: n=15 (2 ineligible; 1 moved away; 12 lost contact)<br><br>Attrition<br>I: n=16 (2 family issues; 2 could not adhere; 2 unrelated health issues; 1 travel; 9 lost contact)<br>C: n=15 (15 lost contact)
		Knee Osteoarthritis		
Strath, 2020 [38]	Food journal to log meals each week (type of food, beverages, sauces, and added sugars/sweeteners and amounts consumed)<br>Adherence: verbally confirmed; food journals assessed by a dietician and the study administrator at each visit	Not measured	NR	LFD: 1 lost to nonadherence<br>C: 2 failed to complete the study

BHB: B-hydroxybutyrate; C: Control; CHO: Carbohydrate; CI: Confidence interval; EORTC QLQ-C30: European Organization for Research and Treatment of Cancer current core; GHS: Global Health Status;; I: Intervention;; KD: Ketogenic diet; LCD: Low-carbohydrate diet; LFD: Low at diet; MCS: Mental component summary; MCT: Medium chain triglycerides; MCTKD: Medium chain triglyceride ketogenic diet; MKD: Modified ketogenic diet; MPD: Modified Paleolithic diet; MUFA: Monounsaturated fatty acids; NR: Not reported; ; NS: Not Significant; PCS: Physical component summary; PUFA: Polyunsaturated fatty acids; QOL: Quality of life; SD: Standard deviation; SFA: Saturated fatty acids; Sig: Significant; T2DM: Type 2 diabetes mellitus.
